# Leucémie aigue érythroblastique: à propos de sept observations

**DOI:** 10.11604/pamj.2014.18.61.3921

**Published:** 2014-05-18

**Authors:** Imane Tlamçani, Salma Benjelloun, Ghita Yahyaoui, Moncef Hassani Amrani

**Affiliations:** 1 Laboratoire d'Hématologie, Laboratoire Central d'Analyses Médicales, CHU Hassan II, Fès, Maroc

**Keywords:** Erythroleucémie, leucémie érythroïde pure, frottis sanguin, médullogramme, imunophénotypage, erythroleukemia, pure erythroid leukemia, blood smear, bone marrow aspiration and biopsy, immunophenotyping

## Abstract

La leucémie aigue érythroblastique (LAM-M6) est une entité rare, représente 3 à 4% de l'ensemble des leucémies aigues. Il en existe deux types: l’érythroleucémie et la leucémie érythroïde pure. Elle se manifeste le plus souvent par des signes de cytopénie et d'infiltration des tissus extra-hématopoïétiques, elle est plus fréquente chez les adultes que chez les enfants et est de mauvais pronostic. Le but de notre travail est de mettre en évidence les particularités épidémiologiques, diagnostiques et évolutives de cette pathologie rare au sein du CHU HASSAN II de Fès. Nous rapportons le cas de sept patients diagnostiqués leucémie aigue érythroblastique LAM-M6 au laboratoire d'hématologie du CHU Hassan II de Fès entre Janvier 2009 et Aout 2013. Le diagnostic de leucémie aigue érythroblastique a été retenu sur un examen cytologique du frottis sanguin et du médullogramme ainsi que l'examen immunophénotypique. Il s'agit de deux adultes et cinq enfants, la plupart ont présenté une altération de l’état général, des signes de cytopénie et un syndrome tumoral. L’étude cytologique du frottis sanguin et du médullogramme ainsi que les résultats de l'immunophénotypage ont conduit au diagnostic de l’érythroleucémie chez six de nos patients et de leucémie érythroïde pure chez un seul patient. L’évolution a été différente pour ces patients. Le pronostic est grave d'où l'intérêt d'un diagnostic rapide et d'une prise en charge adéquate.

## Introduction

La leucémie aigue érythroblastique est caractérisée par la prolifération d'une population érythrocytaire prédominant sur les autres lignées. On en distingue deux types: -érythroleucémie: définie par la présence dans la moelle osseuse de plus de 50% des précurseurs érythroïdes de l'ensemble des cellules médullaires, et de plus de 20% de myéloblastes de l'ensemble des cellules médullaires non érythrocytaires - Leucémie érythroïde pure: elle présente une prolifération néoplasique faite de plus de 80% de cellules érythrocytaires sans présence évidente du contingent myéloblastique.

L’érythroleucémie est une maladie rare, elle représente 3 à 4% des cas de leucémies aigues myéloïdes et est prédominante chez le sujet âgé. Quant à la leucémie érythroïde pure, elle est exceptionnelle et peut concerner tous les âges y compris les enfants [1]. Nous essayons à travers ce travail de mettre le point sur les différentes particularités épidémiologiques, diagnostiques et évolutives de cette pathologie rare au niveau du CHU HASSAN II de Fès et dont le diagnostic a été posé au service d'hématologie du laboratoire central d'analyses médicales.

## Méthodes

Dans cette étude, nous rapportons le cas de sept patients diagnostiqués LAM-M6 au service d'hématologie du CHU Hassan II de Fès entre Janvier 2009 et Aout 2013. Il s'agit de deux adultes de sexe masculin, âgés de 62 et 80ans respectivement, et de cinq enfants d’âges différents allant de 2mois à 8ans. Les patients adultes étaient hospitalisés au service de médecine interne, et les enfants au service de pédiatrie. Nous avons réalisé, pour tous les patients, des frottis sanguins qu'on a colorés à la coloration de May-Grünwald-Giemsa, et des frottis médullaires qu'on a colorés également à la coloration de May-Grünwald-Giemsa et à la myéloperoxydase. Un examen immunophénotypique sanguin et/ou médullaire a été effectué dans tous les cas.

## Résultats

La majorité des patients est issue d'un niveau socio-économique bas et le motif de consultation majeur est l'asthénie associée le plus souvent à un syndrome anémique. L'incidence de la leucémie aigue érythroblastique par rapport aux autres leucémies aigues diagnostiquées au laboratoire d'hématologie du CHU HASSAN II de Fès est de 5%. L’âge moyen de nos patients est de 22,4 ans, et le sexe ratio est H/F = 6 (Tableau 1). Tous les patients présentent une fièvre et une altération d’état général au moment du diagnostic, 85% d'entre eux ont un syndrome tumoral.

**Tableau 1:** Différentes anomalies retrouvées dans les hémogrammes chez tous les patients de notre série

Patient	Age	Sexe	Hb(g/dL)	GB(x109/L)	Plaquettes(x109/)
N°1	62 ans	M	6	19,4	24
N°2	80 ans	M	7,3	83	48
N°3	2 mois	M	5,7	47	33
N°4	2 ans et 6mois	M	5	7,8	44
N°5	3 ans	M	6,4	9,4	27
N°6	3 ans 6mois	F	5,1	6,8	146
N°7	6 ans	M	6,2	2,7	32

Tous les patients présentent une anémie profonde associée à une thrombopénie. Trois des sept patients (enfants) ont un purpura pétéchial à l'admission. L'hyperleucocytose est présente chez trois patients et la leucopénie chez un seul. La fièvre est retrouvée chez tous les patients mais aucun foyer infectieux n'a été mis en évidence (Tableau 1). Le frottis sanguin montre la présence de blastes périphériques dans 71% des cas (Tableau 2).

**Tableau 2:** Différentes anomalies cytologiques retrouvées au niveau des frottis sanguins et myélogrammes chez tous les patients

Patient	Frottis sanguin	Médullogramme
N°1	33% de blastes	25% de myéloblastes+ hyperplasie érythroblastique à 54%+ dysérythropoïèse
N°2	21% d’érythroblastes et 50%de blastes	50% de myéloblastes et de blastes indifférenciés+ lignée érythroblastique à 52%+ signes de mégaloblastose
N°3	Absence de blastes périphériques	28% de blastes+ 63% d’érythroblastes+ dysérythropoïèse
N°4	22% de blastes	25% de myéloblastes+ 52% d’érythroblastes
N°5	5% de blastes	57% de myéloblastes et blastes indifférenciés+ 60% d’érythroblastes+ dysérythropoïèse
N°6	Absence de blastes	14% de myéloblastes+ 82% d’érythroblastes
N°7	3% de blastes	33% de myéloblastes+ 56% d’érythroblastes+ dysérythropoïèse

**Figure 1:**
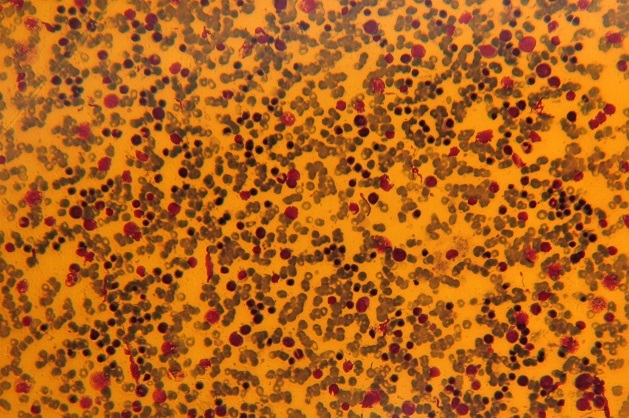
Frottis médullaire montrant une moelle hypercellulaire avec présence de nombreux éléments de la lignée éryhtrocytaire avec différents stades de maturation (objectif x 10 et 20 respectivement)

**Figure 2:**
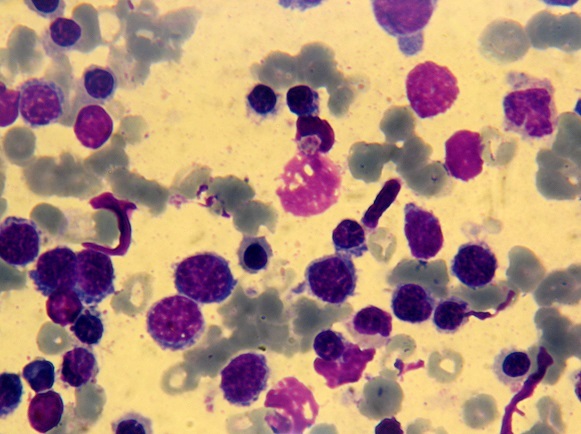
Moelle osseuse hypercellulaire infiltrée par un contingent érythroblastique dépassant 50% de l'ensemble des cellules médullaires avec des signes de dysérythropoïèse (objectif x100)

Dans cette étude, tous les médullogrammes de nos patients répondent aux critères morphologiques établis par la classification FAB (Franco-Américano-Britannique) pour le diagnostic de LAM-M6. Chez six de nos patients, la moelle est hypercellulaire avec un taux de myéloblastes supérieur à 20% par rapport à tous les éléments non érythroblastiques et une hyperplasie érythroblastique estimée à plus de 50%; avec des signes de dysérythropoïèse ce qui a fait évoquer le diagnostic d’érythroleucémie. Un seul patient a une hyperplasie érythroblastique chiffrée à 82% avec 14% de myéloblastes sur les éléments non érythroblastiques, le diagnostic de leucémie érythroïde pure est évoqué (Figure 1, Figure 2, Tableau 2).

**Figure 3:**
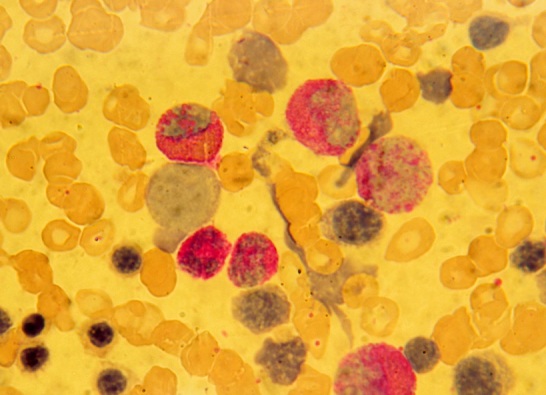
Présence de myéloblastes discrètement positifs à la coloration cytochimique de la myéloperoxydase

Une coloration cytochimique de la myéloperoxydase est réalisée pour tous les médullogrammes et est positive dans tous les cas (Figure 3). L'immunophénotypage sanguin est réalisé pour tous les patients et est positif pour MPO, CD13, CD33 et la Glycophotine A. Quatre de nos patients ont bénéficié d'une étude cytogénétique. Le bilan biochimique a montré un syndrome de lyse tumorale dans tous les cas.

Deux enfants sont décédés au décours du diagnostic. Un patient adulte a refusé le traitement et est décédé après quatre mois suite aux complications. Les autres patients ont été mis sous chimiothérapie, deux d'entre eux sont décédés au cours du traitement. Les deux enfants vivants sont toujours sous traitement et évoluent bien sous chimiothérapie.

## Discussion

La leucémie aigue érythroblastique est une entité très rare. Une anémie importante en est le signe habituel; elle donne lieu à une asthénie profonde. Un syndrome infectieux et/ou hémorragique peuvent être révélateurs. Une hépatomégalie et/ou splénomégalie peuvent être présents [1, 2].

Dans notre série, six de nos patients sont de sexe masculin, la moyenne d’âge est de 22,4 ans avec des extrêmes d’âge de 2 mois et de 80 ans. Cinq de nos patients sont de bas âge y compris un nourrisson de deux mois. La particularité de notre étude réside dans la fréquence élevée des formes pédiatriques de cette pathologie rare contrairement aux cas rapportés dans la littérature où la forme adulte est la plus fréquente [1, 2].

Tous les patients présentent des signes en rapport avec la cytopénie et la présence d'un syndrome tumoral associé. Le diagnostic biologique est posé devant les résultats de la numération formule sanguine, le frottis sanguin, le médullogramme et l'immunophénotypage.

Tous les hémogrammes ont montré la présence d'une bicytopénie (anémie et thrombopénie), trois d'entre eux présentent par ailleurs un purpura pétéchial à l'admission. Trois des sept patients présentent une hyperleucocytose et un seul présente une leucopénie; ce qui rejoint la plupart des cas décrits dans d'autres études [1, 2]. Le frottis sanguin montre la présence de blastes circulants dans 71% des cas. Dans la littérature, ce taux varie entre 37,4% et 85,2% [1, 3].

Au total, la présentation clinique et hématologique de la leucémie aigue érythroblastique est semblable aux tableaux des autres leucémies aigues, cependant la principale différence se situe au niveau de la moelle osseuse et de l'immunophénotypage.

Dans l’érythroleucémie, tous les stades de maturation des précurseurs érythroïdes peuvent être présents avec prédominance des formes immatures. Les précurseurs érythroïdes sont dysplasiques avec éventuellement des formes mégaloblastiques et/ou multinuléées. Le cytoplasme des cellules immatures contient souvent des vacuoles mal limitées qui peuvent fusionner. Les myéloblastes sont de taille moyenne, le cytoplasme contient souvent quelques granulations avec parfois des corps d'Auer. La coloration cytochimique de la myéloperoxydase peut être positive dans les myéloblastes [2, 3].

L’érythroleucémie doit être distinguée des anémies réfractaires avec excès de blastes (AREB) et leucémies aigues myéloïdes (LAM) avec maturation et un taux élevé de précurseurs érythroïdes. Un comptage de toutes les cellules médullaires doit être effectué. Si le taux des précurseurs érythroïdes est supérieur à 50% par rapport à toutes les cellules médullaires, un comptage des cellules non érythroïdes doit être calculé. Si les blastes sont supérieurs à 20% par rapport aux cellules non érythroïdes, le diagnostic est celui de l’érythroleucémie (érythroïde/myéloïde). Si le taux de blastes est inférieur à 20%, le diagnostic est celui de l'AREB [4]. Le diagnostic différentiel inclut également la leucémie aigue myéloïde avec dysplasie multilignée si la dysplasie intéresse plus de 50% des lignées myéloïde ou mégacaryocytaire.

Les érythroblastes ne sont généralement pas associés à des marqueurs myéloïdes et sont négatifs aux anti-MPO, ils réagissent avec des anticorps de la glycophorine A et de l'hémoglobine A. Les myéloblastes expriment une variété d'antigènes myéloïdes associés, y compris: CD13, CD33, CD117 et MPO avec ou sans expression des marqueurs des précurseurs cellulaires, par exemple: CD34 et HLA-DR classe II qui sont des déterminants similaires des blastes d'autres types de LAM [3–5]. Tous les aspects cytologiques décrits dans la littérature sont retrouvés dans notre étude en plus des signes de dysplasie touchant essentiellement la lignée érythrocytaire (<50% de la lignée) et moins fréquemment les lignées mégacaryocytaire et granuleuse [5–7]. La coloration cytochimique de la myéloperoxydase a été positive dans tous les cas ainsi que la présence de marqueurs myéloïdes et érythroblastiques lors de l'immunophénotypage.

Quant à la leucémie érythroïde pure, elle est caractérisée par la présence d’érythroblastes de taille moyenne à grande, souvent à noyau arrondi et à chromatine fine, avec des formes mono et multinucléées. Le cytoplasme est basophile, agranulaire et contenant parfois quelques vacuoles mal limitées [8, 9]. Le diagnostic différentiel de la leucémie érythroïde pure comprend l'anémie mégaloblastique due à une carence en vitamine B12 ou en acide folique [8–10]. Les patients présentant une carence en vitamine B12 et/ou en acide folique répondent au traitement vitaminique, et la dysplasie n'est généralement pas aussi marquée que dans la leucémie érythroïde pure. La segmentation prématurée des précurseurs neutrophiles est une preuve supplémentaire de la carence en vitamine B12 ou en folates [9, 10]. Les formes les plus différenciées peuvent être détectées par l'expression de la glycophorine A et l'hémoglobine A, et l'absence de marqueurs myéloïdes. Les blastes sont souvent négatifs aux anticorps monoclonaux du système HLA-DR classe II et les CD34.

Les formes les plus immatures sont généralement négatives pour la glycophorine A ou faiblement exprimés dans une minorité de blastes. Autres marqueurs tels que l'anhydrase 1 carbonique, CD36 sont habituellement positifs pour les progéniteurs érythroïdes à des stades précoces de leur différenciation. Le CD36 n'est pas spécifique pour les érythroblastes et peut être exprimé par les monocytes et les mégacaryocytes [11]. Les antigènes associés des mégacaryocytes (CD41 et CD61) sont généralement négatifs mais peuvent être partiellement exprimés dans certains cas [12]. Dans notre série, un seul patient est diagnostiqué leucémie érythroïde pure (enfant de trois ans et demi). Au médullogramme, on retrouve une moelle hypercellulaire avec hyperplasie érythroblastique, tous les stades de maturation étant représentés. Le contingent myéloblastique est estimé à 14% compté sur la population non érythrocytaire, fait de blastes de taille moyenne, à noyau arrondi, chromatine fine et souvent nucléolée, cytoplasme peu étendu et basophile contenant parfois des corps d'Auer. La coloration cytochimique à la myéperoxydase est discrètement positive. Quant à la lignée érythroblastique, elle est estimée à 82% faite le plus souvent de mégaloblastes à noyau arrondi et à chromatine fine. L’étude immunophénotypique est positive également pour la Glycophorine A. Le dosage de la vitamine B12 et de l'acide folique est normal ce qui a conclu au diagnostic de LAM-M6 en tenant compte du contexte clinique. Cuneo et al a effectué une étude d'une série de 26 patients avec leucémie aigue érythroblastique et a établi leurs caractéristiques cliniques, morphologiques et cytogénétiques. L'analyse des données de cette étude a montré que lorsque la LAM-M6 est définie par des critères morphologiques, on peut distinguer deux sous groupes [5]: -les patients du premier groupe ont tendance à être plus âgés, avec des anomalies cytogénétiques complexes en particulier des chromosomes 5 et/ou 7, ont un pronostic défavorable. La durée de rémission est courte en utilisant la chimiothérapie conventionnelle. Il est concevable que l'accumulation progressive de l'ensemble de ces altérations génétiques soit importante pour la progression de la maladie de LAM-M6. -Ceux du deuxième groupe ne présentent pas d'anomalies cytogénétiques décelables ou caryotype normal, ont un pronostic plus favorable lorsqu'ils sont traités par une chimiothérapie conventionnelle [5].

Nos patients ayant bénéficié d'une étude cytogénétique ne présentent pas d'aberrations chromosomiques particulières. Le diagnostic a été retenu en se basant sur l'aspect morphologique et immunophénotypique. Dans notre série, l’évolution des patients a été différente; cinq sont décédés au décours du diagnostic et du traitement, et deux évoluent bien sous chimiothérapie conventionnelle. Comparativement à d'autres séries, l’évolution de la leucémie aigue érythroblastique est agressive avec une médiane de survie courte [8, 9].

## Conclusion

Il n'existe pas d'anomalies cytogénétiques spécifiques décrites dans ce type de leucémie. Des caryotypes complexes avec des anomalies de structure sont communs avec atteinte fréquente des chromosomes 5 et 7. Les patients avec des caryotypes complexes ou des anomalies des chromosomes 5 et/ou 7 ont un taux plus élevé de rechute que ceux qui ont des caryotypes normaux. Lors de l'induction de la rémission de la maladie, la durée est plus courte par rapport aux autres sous-types des LAM, c'est un signe de mauvais pronostic. Les patients ayant bénéficié d'une greffe de moelle osseuse ont un taux de rechute moins élevé. La greffe de moelle osseuse peut être discutée lors de la première rémission complète en particulier chez les patients à caryotype complexe avec anomalies chromosomiques [4, 5].
